# Local Anaesthesia

**Published:** 1911-03

**Authors:** W. L. Cooke

**Affiliations:** Columbus, Ga.


// Journal-Record of Medicine

Successor to Atlanta Medical and Surgical Journal, Established 1855, and Southern Medical Record, Established 1870.

OWNED BY THE ATLANTA MEDICAL JOURNAL CO.

Published Monthly

*Official Organ Fulton County Medical Society, State Examining Board, Presbyterian Hospital, Atlanta, Birmingham and Atlantic Railroad Surgeons' Association, Chattahoochee Valley Medical and Surgical Association, Etc.*

EDGAR G. BALLENGER., M. D., Editor.
BERNARD WOLFF, M. D., Supervising Editor.
A. W. STIRLING, M. D., C. M., D. P. H., J. S. HURT, B. Ph., M. D.
GEO. M. NILES, M. D., W. J. LOVE, M. D., (Ala.); Associate Editors.
E. W. ALLEN, Business Manager.

COLLABORATORS

Dr. W. F. WESTMORLAND, General Surgery.
F. W. McRAE, M. D., Abdominal Surgery.
H. F. HARRIS, M. D., Pathology and Bacteriology.
E. B. BLOCK, M. D., Diseases of the Nervous System.
MICHAEL HOKE, M. D., Orthopedic Surgery.
CYRUS W. STRICKLER, M. D., Legal Medicine and Medical Legislation.
E. C. DAVIS, A. B., M. D., Obstetrics.
E. G. JONES, A. B., M. D., Gynecology.
R. T. DORSEY, Jr., B. S. M. D., Medicine.
L. M. GAINES, A. B., M. D., Internal Medicine.
J. N. LeCONTE, M. D., Disease of the Stomach and Intestines.
L. B. CLARKE, M. D., Pediatrics.
EDGAR PAULIN, M. D., Opsonic Medicine.
THEODORE TOEPEL, M. D., Mechano Therapy.
R. R. DALY, M. D., Medical Society.
A. W. STIRLING, M. D., etc., Diseases of the Eye, Ear, Nose and Throat.
BERNARD WOLFF, M. D., Diseases of the Skin.
E. G. BALLENGER, M. D., Diseases of the Genito-Urinary Organs.

VOL. LVI. MARCH, 1911 NO. 12.

### LOCAL ANAESTHESIA.

BY DR. W. L. COOKE, COLUMBUS, GA.

In deciding upon any surgical procedure, one of the first things to be considered is, what anaesthetic shall I use? And, all others things being equal, such as thoroughness in technic, etc., the anaesthetic having the least element of risk should be the one of our choice. Now, in my opinion, a local anaesthetic, that is, one which may be injected into the tissues at the site of operation, fulfills this requirement in contradistinction to the general anaesthetics, such as chloroform and ether. Now, while I would not for one moment be considered as decrying the great boon

*Read before the Chattahoochee Valley Medical and Surgical Association of Columbus, Georgia, Jan 12, 1911.

which these agents have proven to humanity, still we all can but admit that their administration is attended by a certain, though small element of danger. And it is this slight risk which deters an overwhelming majority of sufferers, with such troubles as hernia, hemorrhoids, varicocele, etc., from accepting the almost certain cure which general surgery offers. Therefore, it is incumbent upon us as medical men to find a more attractive proposition in the way of an anaesthetic for these unfortunates, and the local or regional anaesthetics fulfill this requirement in the large majority of cases.

Of the local anaesthetics I have used several, such as cocaine, hydrochlorate, B-Eucaine, Quinine with urea hydrochloride, etc., and, after a fair trial have discarded all the others in favor of the hydrochlorate of cocaine. This drug, when properly used, is thorough in its anaesthetic qualities, and is absolutely devoid of danger. And what do we mean by the proper use of cocaine as an anaesthetic? It is simply using it in solutions of the proper strength, that is, from 1-5 to 1-10 of 1%, having the solution sterile and hot, and injecting it into the tissues at the site of operaion, and not into the spinal cord. To insure sterility, I use cocaine put up in glass bulbs, hermetically sealed, each bulb containing enough sterile cocaine and common table salt, so that when its contents are added to one ounce of water you get a 1-5 of 1% cocaine in normal salt solution, as the normal salt solution is the best medium for presenting the cocaine.

When I first began using this method of anaesthesia, I thought that 1-5 of 1% cocaine was as weak a solution as could be used successfully, but further experience has taught me that 1-10 of 1% can be used just as satisfactorily. Some may say that sterile water will act just as well as such a weak solution of cocaine, and I thought so myself for a while, but my experience has not borne out this supposition, as I have failed in almost every instance to get the proper anaesthesia from sterile water.

And now a word as to the technic of injecting the solution. This must be done properly to insure success, no matter what the operation may be, and for this purpose I have found an all-metal syringe, holding about 2 drams, to be most satisfactory. The needle must be of the smallest gauge possible, and it must be sharp.

Having selected the site of the proposed incision, begin at the end nearest the operator, and inject enough of the solution into the skin to produce a small bleb—introduce the needle at the periphery of this bleb, and make another, and so on until the whole line of incision is completed. This may be done without any more pain than was caused by the first needle prick. The solution must be injected into and not through the skin, as the special sensory nerve terminals are located in the layers of the skin. The skin being thoroughly anaesthetized, some of the solution should be injected into the subcutaneous tissue fascia and muscles. After the skin incision is made the nerve trunks, should any be seen, are to be injected, thus effectually blocking them, and cutting off sensation in their area of distribution. And here I might add that where cocaine is used as an anaesthetic, it is always preferable to administer from 1-4 to 3-8 of a grain of morphine about one-half hour before commencing the operation, to allay the nervousness of the patient, and to counteract any ill effects the cocaine may have, as morphine has been proven to be the physiological antidote to the bad effects of cocaine. This, I say after having used it hundreds of times with no deleterious effect from the cocaine.

I have used as much as 1 1-2 grains of cocaine in one operation, and would not hesitate to use 2 or 3 grains should the occasion demand it.

One factor which enters very largely into determining the success or failure of local anaesthesia is the personal equation of the surgeon himself. A nervous jerky surgeon will undoubtedly produce the same kind of patient, and a nervous, high-strung patient who cannot be controlled, precludes the idea of doing a very extensive operation under local anaesthesia. On the other hand, if the surgeon be possessed of a calm, quiet and positive demeanor and show by his every word and action that he has the utmost confidence in his own ability to do the operation in question, it will go a long way towards inspiring confidence in the patient and will quiet his nerve tension, thus adding very materially to the success of the anaesthesia.

I do not mean by this that the surgeon must be a hypnotist, to do successful work with local anaesthesia, but he must have the power of self-control.

Now, as to the scope of local anaesthesia in general surgery. First, in the opening of an ordinary boil or felon. This looks to be too insignificant an operation for a general anaesthetic, still we all know how terribly painful it is without any anaesthesia, and it can be accomplished without any pain at all, by using a cocaine solution properly. Don't jab the needle right into the abscess to start with, but commence to inject the solution about an inch or more away from the location of proposed incision, in healthy tissue, and gradually extend the infiltration up to the desired point, then the cut may be made slowly and thoroughly with no pain. Another operation for which a general anaesthetic is ordinarily used is the amputation of fingers and toes. However, this may be done very satisfactorily by thoroughly injecting the cocaine solution around the base of the member to be amputated, first into the skin, and then the deeper tissue around the bone, in order to block the nerves. If the solution be properly injected, cuttng through the bone will cause no more pain than the soft tissues. The removal of warty growths, pigmented moles, lipomata, etc., should never require a general anaesthetic, but can be done painlessly with cocaine.

Upon several occasions I have done the operation for hydrocele and varicocele with this anaesthetic, and caused no pain at all. Three times have I done the operation of castration, with the same happy result, so far as pain was concerned. A large majority of goitre operations, both for the simple and exophthalmic variety may be done with this form of anaesthesia.

Twice have I done an inguinal colostomy by this method with no pain at all, when the administration of a general anaesthetic would almost certainly have proven fatal in both cases. Upon several occasions I have drained appendiceal abscesses, without producing either pain or shock. Also I have drained a pelvic abscess per vaginam, after thoroughly infiltrating the tissue back of the cervix. The open operation for fistula in ano may, in a large majority of cases, be done by this method, also the operation for the radical cure of hemorrhoids.

Trachellorhaphy and perineorrphly are amenable to this form of anaesthesia, also a thorough dilatation of the cervix.

A simple cystotomy should hardly ever require a general anaesthetic, and upon one occasion I did an external urethrotomy by this method. It is not within my province to mention the many operations in eye, nose and throat work which may be done under this form of anaesthesia. But it is in the operation for the radical cure of hernia that this form of anaesthesia finds its widest field of usefulness. I have operated about 50 times by this method, for the cure of hernias of the inguinal femoral and umbilical type, and always with the most eminent success, both as to absence of pain and final cure of the trouble. Several times in the course of these operations, I have removed large pieces of omentum, with absolutely no pain or shock. The cure of inguinal hernia lends itself peculiarly to this form of anaesthesia, by reason of the nerve distribution in this locality. The three nerves which supply this region are, in the order of their importance, the ilio-hypogastric, ilio-inguinal, and genital branch of genito-crural. These nerves when found in the course of the dissection, should be injected with the solution, thus effectually blocking them, and rendering their areas of distribution insensitive to pain.

The advantages of this method are most apparent in operating for strangulated hernia. Here we have a patient who is very frequently almost dead to start with, and the administration of a general anaesthetic would be very apt to turn the tide against him. Whereas, with the local anaesthetic, there is no shock whatever, and after the strangulated intestine is released, the patient's condition is improving all the time, so that we need not be in a hurry, but may take as much time as necessary to apply gauze wrung out of hot water to the intestine in the effort to restore its circulation, and thus avoid the necessity of a resection. Several times I have spent as much as thirty minutes carrying out this procedure, and each time have been awarded by seeing the circulation gradually return in a very dark intestine, which I would certainly have resected immediately, had the patient been under a general anaesthetic.

In order to emphasize some of the above statements, I decided to report the following cases:

Case No. 1.—Mr. S., clerk by occupation. Diagnosis—varicocele. Operation in May, 1910. I infiltrated the skin and subcutaneous tissue, then made the incision, exposing the cord and dilated mass of veins. After exposing the cord I thoroughly injected it, in order to block the nerves. The veins were next ligated in two places, about an inch apart, and the intervening section removed. I then incised the parietal portion of tunica vaginalis turned it back and sutured it to the tissues of cord, above the exsected area, thus completely enveloping the ligatures and stumps of veins. The skin sutures were then placed, all of which was done without any pain. Recovery uneventful.

Case 2. Colored woman. Occupation, cook. Diagnosis, simple goitre, about as large as a turkey egg. Operation October 15th, 1910. The skin and subcutaneous tissue were infiltrated and incised, using the transverse collar incision, the platysma was also cut and turned back. Then the capsule of the gland was injected, also some of the solution was placed behind the growth, after which the goitre was removed by a very tedious dissection, during which several large blood vessels were cut and tied, all of which caused no pain whatever. The patient made a complete recovery.

Case No. 3.—Mrs. D., housewife. Diagnosis, lacerated perineum and cervix with subinvolution, operation in Sept. 1910. I first thoroughly infiltrated the cervical tissues, then dilated the cervix and did a curettement. I then repaired the cervix, which was quite extensively lacerated on both sides. After this I repaired the perineum, which was lacerated almost back to the sphincter. Patient remained in bed two weeks, complete recovery.

Case No. 4.—Mr. S., a green countryman, who was very nervous and scary. Diagnosis, left inguinal hernia. Operation Dec. 1st. I transplanted the cord in this case, as is my usual method, and sutured the lower edge of the upper portion of aponeurosis of enternal oblique, along with the conjoined tendon, to Pouparts ligament, them imbricated the lower portion of aponeurosis over the cord, thus making the abdominal wall doubly strong in this region. I have found this step of great advantage in the radical cure of inguinal hernia.

Case No. 5.—Mrs. S., housewife, age about 50, diagnosis, irreducible umbilical hernia. This patient weighed about 250 pounds, and had a bad heart and kidneys, so I would not consider for a moment the administration of a general anaesthetic. I outlined an elliptic incision over site of hernia about 7 inches long by 3 inches wide in the center. This portion of skin and fat, about 2 inches thick, I removed down to the muscles, then opened the sack and removed about a pound of adherent omentum, after which I closed the hernial opening by the overlapping from above down method of the Mayos. This patient made an uneventful recovery, and has had no further trouble. This was done about 18 months ago.

These few cases I have selected at random from my list to illustrate what can really be done with local anaesthesia.

In each case the patient expressed themselves as having had no pain worth mentioning, and that this method was far preferable to being put to sleep. And in four out of five the patients would never have consented to the operation had it not been for the fact that the work could be done with local anaesthesia.

*Discussion of the paper of Dr. W. L. Cooke*:

Dr. Monroe.—I enjoyed the doctor's paper very much, being in Columbus, and seeing the result of his work along this line, I think he is to be congratulated on the good work he has done. He has performed a good many operations where the people would not take a general anaesthetic. I do not speak against general anaesthesia, but the public is afraid of it, and when we can do an operation with a local anaesthesia, it is preferable. In regard to the choice of anaesthetics, I have been using quinine and urea, and I think it preferable to cocaine, as under certain conditions it acts better than cocaine, and there is less danger of poison. Dr. Cooke does not seem to fear the symptoms of poisoning from cocaine, but sometimes a very small dose gives symptoms of poison. I agree with him about the use of morphine before injecting cocaine. The main advantage of quinine and urea is that it lasts longer, and in operations on the anus, for instance, the after pain is great, and thus a longer anaesthetic is needed. It takes longer to get the effects of quinine and urea, but after getting it, the anaesthesia is more complete. It does give a little thickening to the edges of the wound, therefore, does not heal as rapidly as where cocaine is used. Dr. Cooke's idea about the operation for hernia is correct, and I can vouch for every word of it, as I have had personal experience in this operation, with good results. I have found in hydrocele operations, local anaesthesia is not so effective. I certainly enjoyed the paper, and think each member can take every word of it to heart, and you will find the time is coming when local anaesthesia will be largely used.

Dr. C. L. Williams.—These two gentlemen have spoken rather lightly of the chloroform anaesthesia, they say there is very little danger, while in my opinion, and from experience, it is a very difficult matter to tell when chloroform is dangerous. I have seen patients on which this was used, where the operation had to be abandoned as they could not stand it. I have seen fatal results from both chloroform and ether, and from ether, I have always been exceedingly careful in giving anaesthetics.

Dr. J. L. B. Branch of Macon asked Dr. Cooke if he had ever used adrenalin with cocaine.

Dr. Bowman.—The doctor mentioned the fact of pain making the patient sick. I have often wondered if the effects attributed to cocaine are not often from pain. One case especially I remember, a patient with a boil on his neck, where I used cocaine, and made an incision without pain, and endeavored to make incision deeper and caused great pain, and my patient fell over, as I thought, from the effects of the cocaine, had all the symptoms, so I put him to bed, gave the morphine, etc. The next day in treating the wound I caused much pain, and the patient experienced all the symptoms which, on the previous day I had attributed to cocaine.

Dr. Cooke, in closing.—I want to thank the gentlemen for their criticism and endorsement of my paper. One thing I want to add is as to the sterilizing of cocaine. In my opinion if you boil the cocaine solution, you will almost destroy the strength of the cocaine. In my paper I mentioned the fact that I got the cocaine in glass tubes, sterilized the tube, and then broke the tube and emptied the solution into warm water. In using cocaine, I have had a little pain where I have had time to inject it properly, but otherwise have had no pain at all, although I have explored the whole perineum, and repaired the perineum which was lacerated almost back to the sphintcer. Also repaired the cervix. I have never used adrenalin with cocaine, as I have always gotten good results without it. I have never had any symptoms of poison that it could attribute to cocaine, as I have always used morphine about one-half hour before using the cocaine. Upon a few occasions, where I have not used morphine, got some symptoms which I thought due to nervous shock, and not due to cocaine. As to the use of quinine and urea, I have tried it on several occasions, but possibly did not use it strong enough, as I did not get the anaesthetic effect I expected.

